# Factors associated with exclusive breastfeeding of children under six months of age in Cote d’Ivoire

**DOI:** 10.1186/s13006-023-00573-1

**Published:** 2023-08-15

**Authors:** Ibrahima Koffi, Esme Marie Laure Essis, Iba Bamba, Kaudjhis Rh Assi, Loukou Léandre Konan, Joseph Aka

**Affiliations:** 1 Directorate of Strategy and Studies, Ministry of Planning and Development, Abidjan, Côte d’Ivoire; 2 Center for Population and Health Policy and Systems Research, National Institute of Public Health, Abidjan, Côte d’Ivoire; 3 Cellule de Recherche en Santé de la Reproduction de Cote d’Ivoire, Abidjan, Côte d’Ivoire; 4 Nutrition Department National Institute of Public Health, Abidjan, Côte d’Ivoire; 5 Department of Public Health and Biostatistics, UFR of Medical Sciences, Faculty of Medicine, Félix Houphouët Boigny University, Abidjan, Côte d’Ivoire

**Keywords:** Exclusive breastfeeding, Duration, Infant, Explanatory factors, Abidjan, Cote d’Ivoire

## Abstract

**Background:**

Despite benefits of exclusive breastfeeding (EBF) and its strategic place in the national guidelines for infant and young child feeding, its practice remains insufficient in Cote d’Ivoire. It is therefore important to identify its early stopping associated factors. We aimed to (i) assess the extent of children’s exposure to exclusive breastfeeding and the associated explanatory factors for discontinuation before six months, and (ii) to profile non-exclusively breastfed children and interrelationships between these factors.

**Methods:**

A secondary analysis of data from the 2016 Cote d’Ivoire Fifth Multi Indicator Cluster Survey (MICS5) of 980 children under six months of age was conducted in this study. Data were analyzed using the actuarial method of survival hazard estimation combined with the Wilcoxon (Gehan) test, discrete time proportional hazards regression models, and Multiple Correspondence Analysis (MCA) to profile the children.

**Results:**

Maternal exposure to counseling session, age at delivery, and child sex were significantly associated with the likelihood of discontinuing exclusive breastfeeding before the first six months of life. Children deprived of EBF resided in urban areas, in high and very high economic welfare households. Their mothers had a secondary education or higher and had three or fewer children.

Logistic analysis showed that health status and sex of the child were significantly associated (*P* < 0.001) with exclusive breastfeeding. An extremely important and rarely studied factor is that children who were sick in weeks prior to the survey were more likely to remain exclusive breastfeeding (adjusted OR 1.80; 95% Confidence Interval (CI) 1.452, 2.234). Girls are less likely to be exclusively breastfed than boys (adjusted OR 1.48; 95% CI 1.22, 1.798). Low standard of living was associated with early cessation of EBF (adjusted OR 2.15; CI 1.325, 3.499). The duration of the exclusive breastfeeding was significantly longer among mothers with high exposure to medical discourse (adjusted OR 0.74; CI 0.595, 0.91).

**Conclusions:**

Improving the practice of exclusive breastfeeding in Cote d’Ivoire requires strengthening the capacities of health professionals in terms of advice and assistance to mothers for the practice of exclusive breastfeeding and its maintenance until six months of age, regardless of the health status and sex of the child.

## Background

Child health remains an important issue in sub-Saharan Africa and national and international policymakers are looking for strategies to improve it [1]. Among the strategies identified to positively impact child health and reduce infant and child mortality, the promotion of breastfeeding occupies a predominant place [2, 3]. The benefit of exclusive breastfeeding (EBF) is clear at the social and economic level and can significantly reduce infant mortality [4]. If breastfeeding were to become an almost universal practice, 823,000 deaths of children under five years of age would be prevented each year and the annual number of deaths from breast cancer would be reduced by at least 20,000 cases. It would also prevent ovarian cancer, type 2 diabetes, and several childhood diseases such as diarrhea, acute respiratory infections, obesity, etc. [5, 6]. It also facilitates optimal and harmonious growth throughout the child’s physical and cognitive development [7]. In recognition of the critical role of appropriate infant feeding, WHO and UNICEF recommend that infants be exclusively breastfed for the first six months of life for optimal growth, development, and health. After six months, as their nutritional needs change, infants should be fed safe and nutritionally adequate complementary foods while continuing to be breastfed for up to two years or more [3, 8].

In Cote d’Ivoire, in 2021, the exclusive breastfeeding rate which referred to the proportion of infants 0–5 months of age who are fed exclusively with breast milk was 34% according to the Demographic and Health Survey [9]. Although it has increased since 2006, it’s still below the objective that the Ivorian government set in its National Strategic Plan for Mother, Newborn and Child Health, i.e., to reach a rate of 50% by 2020 [10]. This low rate makes Cote d’Ivoire one of the West African countries with the lowest EBF prevalence, and more than seven out of 10 mother-child pairs miss out on the benefits of exclusive breastfeeding. This low practice compromises achievement of the national nutrition and health development policy objectives insofar as it contributes to maintaining maternal and child morbidity and even infant and child mortality at a relatively high level.

Although work has been done on the issue of breastfeeding in Cote d’Ivoire [11, 12], none to our knowledge has looked at factors related to the initiation and duration of exclusive breastfeeding based on a nationally representative sample. Our study addresses the issue of exclusive breastfeeding from the perspective of factors influencing its duration. The results of this study may contribute to improving knowledge about exclusive breastfeeding and help policymakers implement strategies to reach the 50% target by 2025.

Our main objective is to gain a better understanding of the factors influencing the duration of exclusive breastfeeding in Cote d’Ivoire, with a view to improving maternal and child health. The aim is to describe variations in the duration of exclusive breastfeeding according to certain characteristics, to draw up a profile of children deprived of exclusive breastfeeding, and to identify the factors influencing the duration of exclusive breastfeeding.

## Methods

### Data source

Data for this study come from the 2016 Fifth Multiple Indicator Cluster Survey (MICS-5) conducted by the National Institute of Statistics in collaboration with UNICEF and other partners. This is a cross-sectional, nationally representative survey covering 3390 children under two years of age. This analysis involved 980 children aged 0 to 5 months.

### Definition of EBF and indicator used on this study

According to WHO, exclusive breastfeeding is defined as giving no other food or drink, not even water, except breast milk. It does, however, allow the infant to receive oral rehydration salts (ORS), drops and syrups (vitamins, minerals, and medicines). It includes breastfeeding by a wet nurse and feeding expressed breast milk [13].

Exclusive breastfeeding under six months is the indicator used. This indicator referred to the proportion of infants 0–5 months of age who are fed exclusively with breast milk. It is a current status indicators based on recall of the previous day (24 h) before a questionnaire was administered to mothers and involved only living children [13].

### Explanatory variables

The independent variables of our study were selected based on a review of the literature and elements of the Ivorian social context. The different concepts and operational variables of the study are summarized in Table 1.

**Table 1:** Study concepts and operational variables

Concepts		Operational variables
Contextual characteristics	Residence context	Region of residence
		Place of residence
	Sociocultural context	Religion
		Ethnicity
	Socioeconomic characteristics of the household	Household standard of living
		Cohabitation of the partners
Individual characteristics of mother	Economic characteristics	Occupation
	Biodemographic characteristics	Age at delivery
		Parity
	Sociocultural characteristics	Education level
		Media exposure
Individual characteristics of the child		Sex
		Health status
		Desirability (importance of the child to the mother)

Some independent variables were constructed by combining two or three variables. These are the following variables:

### Exposure to medical discourse on breastfeeding

This was constructed from a combination of variables such as the prenatal consultation, the number of consultations and the quality of the person who attended the birth. We assigned the following modalities: (1) *Non-exposed*: those who had never attended a prenatal consultation and who had not been assisted by a qualified person during their delivery; (2) *Highly exposed*: those who had attended more than three prenatal consultations and had been assisted by a qualified person during their delivery; and (3) *Lowly exposed*: those who fell between these two modalities.

### Exposure to the media

This was constructed from two variables: frequency of radio listening and frequency of television viewing. The variable ‘frequency of reading a newspaper or magazine’ was not used because it had a non-response rate of over 10%. Three modalities were defined: (1) *Not exposed*: concerns women who do not follow radio and television at all; (2) *Moderately*: those who often follow one or both media and (3) *Higher*: those who are exposed to both media every day.

**Fig. 1:**
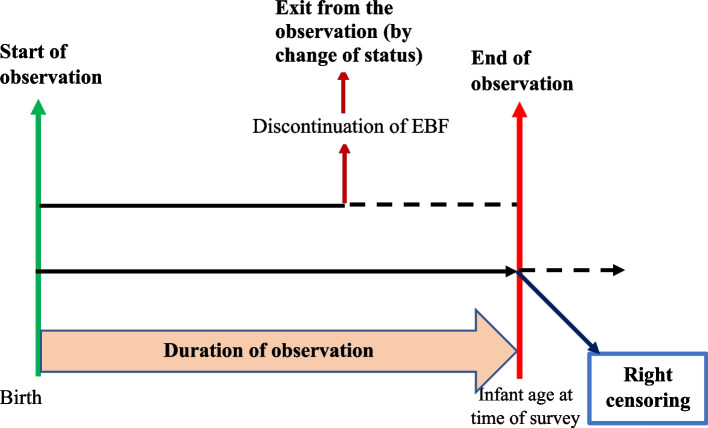
Illustration of the different types of outputs or truncations

The dependent variable of the study was the duration of the exclusive breastfeeding, that was conceptualized as described below (Fig. 1).

### Conceptualization of the event studied

The conceptualization consists in defining the event under study, the population at risk, as well as the competing events that may cause the individual to leave the observation, with or without a change in state [14] .

- **Event under study**: discontinuation of EBF before six months.
- **Population at risk**: children under six months of age at the time of the survey.
- **Start date of observation**: children’s date of birth.
- **End date of observation**: date on which the child ate or drank anything other than breast milk. The duration of observation is conditioned by two types of discharge: discharge from **observation without change of state and discharge with change of state.**
- **Exit without change of condition (right censoring)**: child exclusively breastfed at the date of the survey. The child’s end of observation date will be the date of the survey. It is then considered right censored.
- ** Exit by change of status**: child having eaten or drunk food other than breast milk which interrupts the exclusive breastfeeding before the survey date.

These two observations exit (event occurrence and survey date) are the only ones considered in this study.

- **Scope of the study**: the entire Ivorian territory covered by the MICS 2016 survey.
- **Duration of observation**: This is the time that elapses from the date of birth to the date of cessation of exclusive breastfeeding and from the date of birth to the date of the survey. This corresponds, for the first type, to the age of the child at the time EBF was stopped (date of consumption of a food other than breast milk - date of birth) and, for the second, to the age of the child at the time of the survey (date of the survey - date of birth). (Fig. 1).

**Unit of time:** the month.

### Methods of analysis

Discrete time Survival Analysis were used. At the descriptive level, the actuarial method of estimating the survival hazard associated with the Wilcoxon (Gehan) test at the 5% threshold and, at the explanatory level, discrete-time logistic regression. A Multiple Correspondence Analysis (MCA) was used to profile children according to whether they were exclusively breastfed or not at the time of the survey.

At the explanatory level, several stepwise discrete time proportional hazards regression models were constructed to highlight the interrelationships between the different variables entered in the regression according to the analysis scheme. The odds ratios for the propensity to stop exclusive breastfeeding are presented in Table 2. The different models selected are as follows:

**Table 2:** Results (Odds ratios) of discrete-time logistic regression models: crude and net effects of independent variables on the risk of stopping exclusive breastfeeding

Variables and modalities	Crude effects	Net effects													
		M1	M2	M3	M4	M5	M6	M7	M8	M9	M10	M11	M12	M13	M14
Region of residence	*	***	***	***	***	***	***	***	***	***	***	***	***	***	***
East	0.753^ns^	0.503**	0.497**	0.478***	0.483***	0.492**	0.479***	0.485**	0.476***	0.477***	0.491**	0.508**	0.544**	0.544**	0.544**
West	0.837^ns^	0.625***	0.620***	0.602***	0.628***	0.625***	0.625***	0.622***	0.603***	0.603***	0.638***	0.627***	0.634***	0.647***	0.647***
Central	0.926^ns^	0.854^ns^	0.881^ns^	0.800^ns^	0.800^ns^	0.738^ns^	0.724*	0.728*	0.733^ns^	0.736^ns^	0.808^ns^	0.812^ns^	0.845^ns^	0.787^ns^	0.787^ns^
North	1	1	1	1	1	1	1	1	1	1	1	1	1	1	1
South without Abidjan	1.025^ns^	0.950^ns^	0.931^ns^	0.866^ns^	0.860^ns^	0.884^ns^	0.883^ns^	0.892^ns^	0.877^ns^	0.880^ns^	0.935^ns^	0.935^ns^	0.962^ns^	0.931^ns^	0.931^ns^
City of Abidjan	1.230^ns^	1.122^ns^	1.354^ns^	1.264^ns^	1.279^ns^	1.636**	1.643**	1.617**	1.594**	1.593**	1.796**	1.875***	2.078***	2.002***	2.000***
Place of residence	ns		**	**	**	ns	ns	ns	ns	ns	ns	ns	*	ns	ns
Urban	1.108^ns^		0.774**	0.785**	0.743 ^**^	0.803^ns^	0.801^ns^	0.790^ns^	0.787^ns^	0.787^ns^	0.783^ns^	0.794^ns^	0.770*	0.778^ns^	0.778^ns^
Rural	1		1	1	1	1	1	1	1	1	1	1	1	1	1
Mother's ethnicity	ns			ns	ns	ns	ns	ns	ns	ns	ns	ns	ns	*	*
Akan	1.119^ns^			1.225^ns^	1.419**	1.497**	1.418**	1.439**	1,49**	1,493**	1,487**	1,470**	1,498**	1,561**	1,560**
Krou	1.105^ns^			1.065^ns^	1.23^ns^	1.413^ns^	1397^ns^	1.494^ns^	1,561*	1,572*	1,614*	1,614*	1,657*	1,658*	1,657*
Mandé	1.030^ns^			1.043^ns^	1.08ns	1.140^ns^	1.123^ns^	1.130^ns^	1.153^ns^	1,154^ns^	1,197^ns^	1,210^ns^	1,229^ns^	1,292^ns^	1,291^ns^
Gur	0.981^ns^			1.002^ns^	1.068^ns^	1.089^ns^	1.083^ns^	1.069^ns^	1.068^ns^	1.066^ns^	1.053^ns^	1.061^ns^	1.082^ns^	1.067^ns^	1.067^ns^
Foreign/ Other	1			1	1	1	1	1	1	1	1	1	1	1	1
Religion	ns				ns	ns	ns	ns	ns	ns	ns	ns	ns	ns	ns
Muslim	Ref				1	1	1	1	1	1	1	1	1	1	1
Catholic	0.904^ns^				0.824^ns^	0.826^ns^	0.819^ns^	0.815^ns^	0.832^ns^	0.829^ns^	0.849^ns^	0.865^ns^	0.813^ns^	0.836^ns^	.836^ns^
Other Christians	1.028^ns^				0.85^ns^	0.848^ns^	0.857^ns^	0.841^ns^	0.854^ns^	0.851^ns^	0.844^ns^	0.874^ns^	0.848^ns^	0.915^ns^	.914^ns^
Animist/Other	0.879^ns^				0.742*	0.755^ns^	0.756^ns^	0.745*	0.739*	0.739*	0.705*	0.725*	0.726*	0.711*	0.711*
Household standard of living	ns					***	***	***	***	***	***	***	***	***	***
Very low	0.985^ns^					1.754**	1.799**	1.876***	1.844**	1.839**	1.583*	1.550*	1.473ns	1.587*	1.587*
Low	1.164^ns^					2.197***	2.215***	2.297***	2.246***	2.246***	2.063***	1.991***	1.948***	2.154***	2.153***
Medium	1.089^ns^					1.808***	1.836***	1.873***	1.866***	1.866***	1.765**	1.731**	1.753**	1.849***	1.848***
High	1.365**					2.317***	2.382***	2.458***	2.488***	2.490***	2.312***	2.299***	2.248***	2.536***	2.534***
Very high	1					1	1	1	1	1	1	1	1	1	1
Cohabitation of spouses	ns						ns	ns	ns	ns	ns	ns	ns	ns	ns
With spouse	0.872^ns^						0.845^ns^	0.830^ns^	0.911^ns^	0.912^ns^	0.897^ns^	0.944^ns^	0.946^ns^	0.893^ns^	0.894^ns^
Alone	1						1	1	1	1	1	1	1	1	1
Level of education	ns							ns	ns	ns	ns	ns	ns	**	**
None	0.914^ns^							0.835^ns^	0.874^ns^	0.878^ns^	0.838^ns^	0.808^ns^	0.802^ns^	0.725*	0.725*
Primary	0.839^ns^							0.695^ns^	0.726*	0.728*	0.716*	0.687**	0.690**	0.640**	0.639**
Secondary and above	1							1	1	1	1	1	1	1	1
Age at delivery	ns								**	ns	ns	*	*	*	*
Less than 20 years old	1								1	1	1	1	1	1	1
20 - 34 years old	0.863^ns^								0.843^ns^	0.849^ns^	0.850^ns^	0.841^ns^	0.820^ns^	0.797^ns^	0.798^ns^
35 years and older	0.779*								0.620**	0.621**	0.623**	0.597**	0.576**	0.596**	0.597**
Parity	ns									ns	ns	ns	ns	ns	ns
Low	1									1	1	1	1	1	1
Medium	0.958^ns^									0.965^ns^	0.961^ns^	0.971^ns^	1.003^ns^	0.981^ns^	0.980^ns^
High	0.983^ns^									1.005^ns^	1.002^ns^	1.005^ns^	1.009^ns^	1.001^ns^	1.000^ns^
Exposure to medical discourse	ns										***	***	***	***	***
Not exposed	1.114^ns^										1.368^ns^	1.354^ns^	1.372^ns^	1.364^ns^	1.364^ns^
Weakly	1										1	1	1	1	1
Strongly	0.895^ns^										0.734***	0.740***	0.742***	0.736***	0.736***
Exposure to the media	ns											ns	ns	*	*
Not exposed	0.999^ns^											1.225^ns^	1.214^ns^	1.211^ns^	1.210^ns^
Moderately	1.108^ns^											1.374**	1.372**	1.394**	1.393**
More exposed	1											1	1	1	1
Child’s sex	**												***	***	***
Male	1												1	1	1
Female	1.179**												1.451***	1.483***	1.482***
Child's health	**													***	***
Sick	1													1	1
Not ill	1.179**													1.800***	1.801***
Desirability of the child	ns														ns
Desired	0.934^ns^														0.994^ns^
Not desired	1														1
Chi2		629.774	634.479	637.318	640.782	661.850	663.284	667.816	675.322	675.413	687.308	691.892	706.557	736.969	736.972
Pr>Chi2		***	***	***	***	***	***	***	***	***	***	***	***	***	***

NB: Each regression model included 980 children under 6 months of age

Explanatory note: *** = Significant at the 1% level; **= Significant at the 5% level; *= Significant at the 10% level; ns = Not significant;

Model 0 (M0): Child age group (control variable).

Model 1 (M1): Mother’s region of residence.

Model 2 (M2): M1 + Mother’s residence area.

Model 3 (M3): M2 + Mother’s ethnicity.

Model 4 (M4): M3 + Mother’s Religion.

Model 5 (M5): M4 + Household standard of living.

Model 6 (M6): M5 + Spousal Cohabitation.

Model 7 (M7): M6 + Mother’s education level.

Model 8 (M8): M7 + Mother’s age at delivery.

Model 9 (M9): M8 + Parity achieved.

Model 10 (M10): M9 + Exposure to medical discourse on breastfeeding.

Model 11 (M11): M10 + Mother’s exposure to media.

Model 12 (M12): M11 + Child’s sex.

Model 13 (M13): M12 + Child’s health status.

Model 14 (M14): M13 + Child’s Desirability.

The M0 model contains the age of the children introduced as the control variable. The last model (M14) is the saturated model that identifies the net weight of each explanatory variable in the presence of all other variables. The intermediate models (M1 to M13) allow us to detect the interrelations between the different variables. The “categorical” option of the SPSS 25 software was used in the discrete-time logistic regression to simultaneously give the significance of the variables and the modalities.

In order to identify the explanatory variables that contribute most statistically to the model, a hierarchical ranking of the variables was done using the Chi-square of the saturated or final model (${X}_{f}^{2}$). This approach is justified by our desire to prioritize the most relevant interventions to be implemented in our context of budgetary constraints. It consists of ordering the independent variables according to the percentage of Chi-square that they contribute to the explanation of the dependent variable.

Let ${C}_{i}$ be the contribution of the independent variable ***i*** to the explanation of the phenomenon studied, ${\varvec{X}}_{\varvec{f}}^{2}$ the Chi-square of the final model and ${\varvec{X}}_{\varvec{f}-\varvec{i}}^{2}$ the Chi-square without the independent variable ***i***. The contribution of an independent variable ***i*** is given by the following formula:

$${C}_{i}=\frac{{X}_{f}^{2}-{X}_{f-i}^{2}}{{X}_{f}^{2}}$$

## Results

**Fig. 2:**
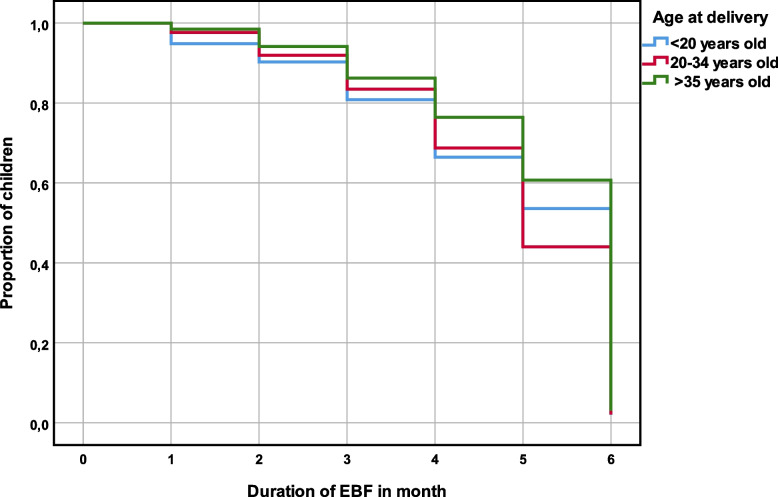
Prevalence of EBF 0–6 months by age of mother at delivery

The national exclusive breastfeeding rate was 23.5% according to the 5th MICS (2016) in Cote d’Ivoire (15). The descriptive analysis involved 980 children under six months of age. The results indicate that maternal age at delivery, exposure to medical discourse on breastfeeding (a composite variable combining the number of antenatal cares and assistance at delivery by skilled personnel), and the child’s sex were significantly associated with the duration of exclusive breastfeeding. Regarding the mother’s age at delivery, after one month of life, the EBF prevalence curves differ significantly from each other until the end of the 6th month (Fig. 2). Moreover, the differences increased over time. The curve representing mothers aged 35 years or more remains above the others throughout the observation period and that of mothers under 20 years of age remains below them (except for the 6th month). This means that children born to older mothers experienced cessation of exclusive breastfeeding later than children born to younger mothers.

**Fig. 3:**
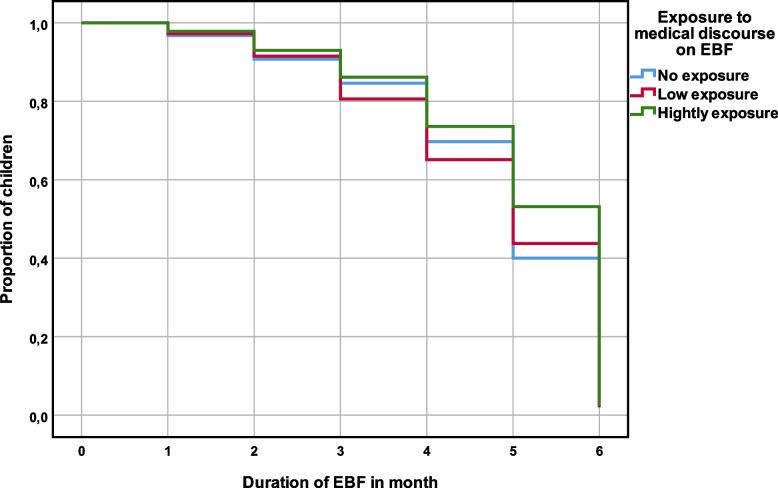
Prevalence of EBF 0–6 by exposure to medical discourse on breastfeeding

Regarding exposure to counseling on breastfeeding or the number of antenatal cares and the qualification of the person who assisted the woman at delivery, we note that the proportion of exclusively breastfed children remains higher among children whose mothers have achieved more than three antenatal cares and had benefit from a skilled attendant at delivery (Fig. 3).

Otherwise, the proportion of children who were fed other foods remained lowest among mothers with high exposure to counseling session on breastfeeding. Thus, children whose mothers had achieved more than three antenatal cares and were assisted by a skilled attendant at delivery were experienced cessation of exclusive breastfeeding less quickly over the 6-month period than other children.

**Fig. 4:**
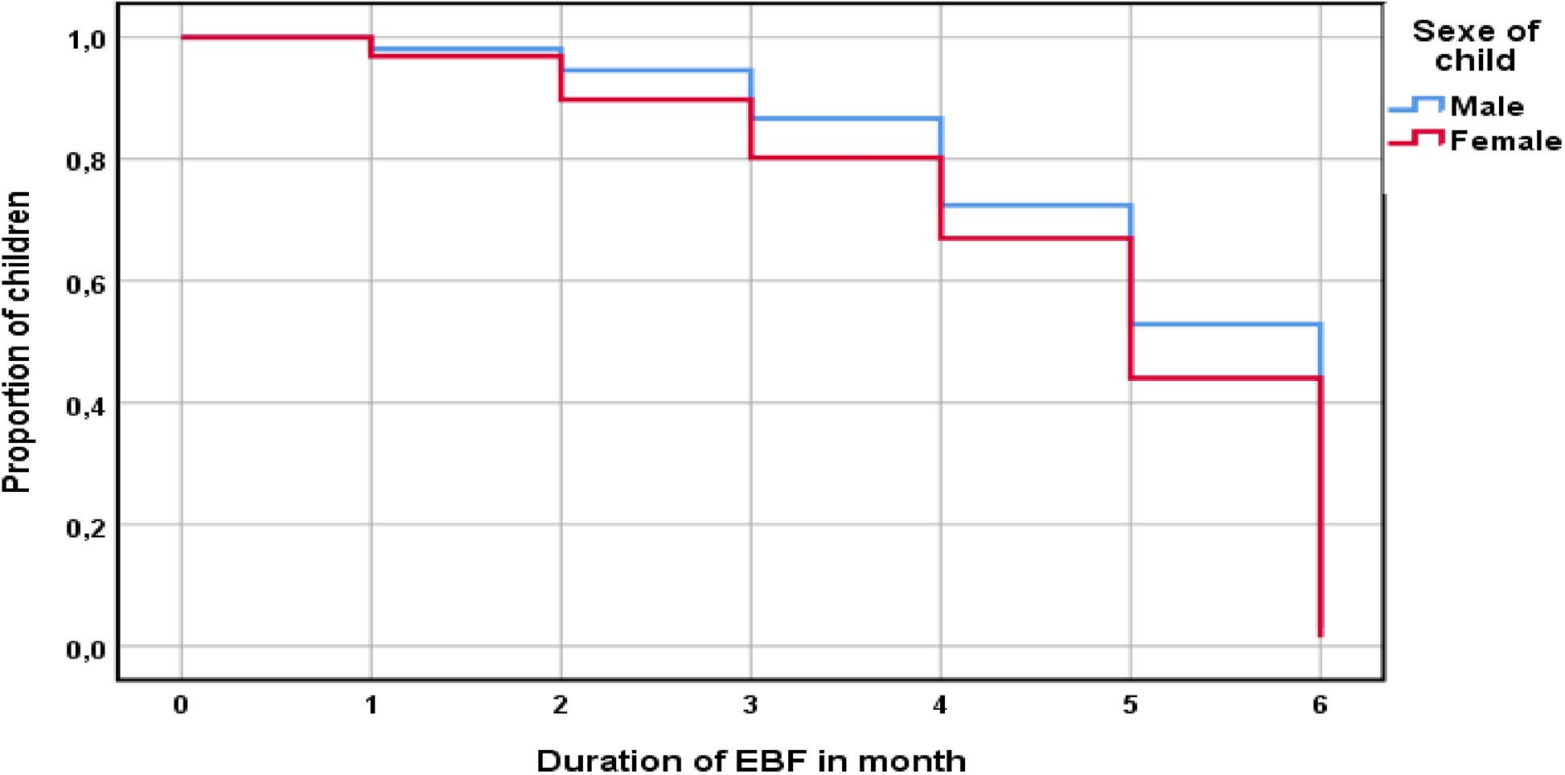
Prevalence of EBF 0–6 by sex of child

Virtually over the entire duration of exposure to exclusive breastfeeding cessation, the prevalence curve for male children is above that for female children. This means that male children are less at risk of stopping EBF than female children (Fig. 4).

**Fig. 5:**
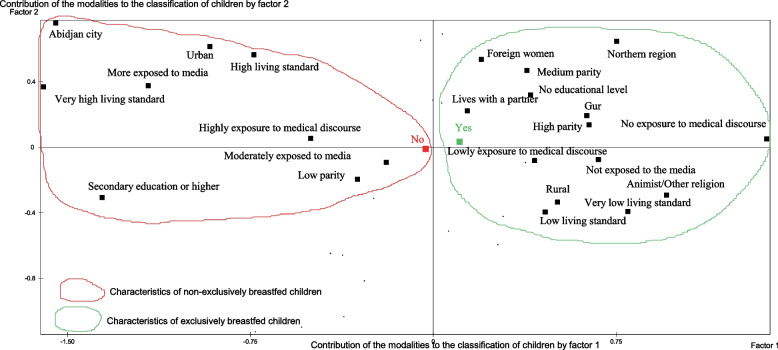
Profile of children aged 0-6 months by EBF status

The Multiple Correspondence Analysis (MCA) was used to profile the children. Factor analysis seeks to reduce a large amount of information (in the form of values on variables) to a few large dimensions (factors). It attempts to decompose the patterns of correlations to explain them by a limited number of factors. Regarding the profile of children (Fig. 5), the MCA identified two groups of children. The target group was composed exclusively of non-exclusively breastfed children. They lived in Abidjan and in urban areas in general. They live in households with high economic well-being. Their mothers had a secondary education or higher and were highly exposed to the media. They had achieved more than three antenatal cares and had been assisted by a skilled attendant at delivery and they have no more than three children.

The second group consists of exclusively breastfed children whose mothers lived in the northern region and in rural areas in general. These women are largely of the Gur ethnic group and from neighboring countries. They belonged to households with low economic well-being with more than four children. They were uneducated, with little or no exposure to breastfeeding counseling and usually live with their partners.

The results of the discrete-time logistic models are presented in Tables 2 and 4. The values provided by these tables are odds ratios but the Table 4 present crude OR, adjusted OR and the 95% CI. The regression models consider 980 children under six months. Child age was a control variable for each model. The Table 3 highlights the explanatory factors of the EBF discontinuation, ranked in decreasing order of their explanatory power: region of residence, child’s health status, household standard of living, child’s sex, mother’s exposure to breastfeeding counseling and mother’s level of education (Tables 3 and 4).

**Table 3:** Hierarchy of explanatory factors for EBF discontinuation in decreasing order of contribution

Explanatory factors	Chi2	Contribution	
	Loss of chi-square value if this variable is removed from the model	Absolute (%)	Relative (%)
Region of residence	736.97, 676.36	8.22	20.33
Child's health status	736.97, 679.95	7.74	19.13
Household standard of living	736.97, 680.14	7.71	19.06
Child's sex	736.97, 691.17	6.21	15.36
Exposure to medical discourse	736.97, 696.9	5.44	13.44
Mother's education level	736.97, 699.18	5.13	12.68

**Table 4:** Determinants of duration of exclusive breastfeeding

Variables	Crude OR (95% CI)	P>|z|	Adjusted OR (95% CI)	P>|z|
**Region of residence**	*		***	
East	0.753 (0.481, 1.181)	0.217	0.544 (0.309, 0.956)	0.034
West	0.837 (0.655, 1.069)	0.154	0.647 (0.469, 0.894)	0.008
Central	0.926 (0.689, 1.245)	0.611	0.787 (0.536, 1.156)	0.222
North	1		1	1
South without Abidjan	1.025 (0.799, 1.315)	0.845	0.931 (0.659, 1.315)	0.686
City of Abidjan	1.230 (0.93, 1.628)	0.148	2 (1.245, 3.214)	0.004
**Place of residence**	ns		ns	
Urban	1.108 (0.938, 1.308)	0.229	0.778 (0.570, 1.061)	0.113
Rural	1		1	1
**Mother's ethnicity**	ns		*	
Akan	1.119 (0.901, 1.39)	0.309	1.560 (1.093, 2.227)	0.014
Krou	1.105 (0.77, 1.584)	0.588	1.657 (0.986, 2.785)	0.057
Mande	1.03 (0.818, 1.297)		1.291 (0.95, 1.757)	0.104
Gur	0.981 (0.771, 1.249)	0.878	1.067 (0.776, 1.467)	0.692
Foreign/ Other	1		1	1
**Religion**	ns		ns	
Muslim	1		1	1
Catholic	0.904 (0.699, 1.169)	0.442	0.836 (0.585, 1.196)	0.327
Other Christians	1.028 (0.841, 1.257)	0.787	0.914 (0.652, 1.282)	0.602
Animist/Other	0.879 (0.678, 1.139)	0.328	0.711 (0.498, 1.016)	0.061
**Household standard of living**	ns		***	
Very low	0.985 (0.75, 1.293)	0.913	1.587 (0.949, 2.654)	0.078
Low	1.164 (0.882, 1.536)	0.283	2.153 (1.325, 3.499)	0.002
Medium	1.089 (0.819, 1.447)	0.558	1.848 (1.189, 2.873)	0.006
High	1.365 (1.005, 1.853)	0.047	2.534 (1.69, 3.8)	0.000
Very high	1	1	1	1
**Cohabitation of spouses**	ns		ns	
With spouse	0.872 (0.701, 1.085)	0.220	0.894 (0.66, 1.209)	0.466
Alone	1	1	1	1
**Level of education**	ns		**	
None	0.914 (0.721, 1.16)	0.46	0.725 (0.516, 1.018)	0.063
Primary	0.839 (0.631, 1.116)	0.228	0.639 (0.447, 0.914)	0.014
Secondary and above	1	1	1	1
**Age at delivery**	ns		*	
Less than 20 years old	1	1	1	1
20 - 34 years old	0.863 (0.68, 1.096)	0.226	0.798 (0.587, 1.085)	0.150
35 years and older	0.779 (0.579, 1.047	0.098	0.597 (0.375, 0.95)	0.030
**Parity**	ns		ns	
Low	1	1	1	1
Medium	0.958 (0.78, 1.177)	0.683	0.980 (0.751, 1.279)	0.884
High	0.983 (0.793, 1.219)	0.875	1 (0.702, 1.425)	1.000
**Exposure to medical discourse**	ns		***	
Not exposed	1.114 (0.866, 1.189)	0.237	1.364 (0.873, 2.133)	0.173
Weakly	1	1	1	1
Strongly	0.895 (0.945, 1.32)	0.193	0.736 (0.595, 0.91)	0.005
**Exposure to the media**	ns		*	
Not exposed	0.999 (0.784, 1.256)	0.993	1.21 (0.859, 1.704)	0.276
Moderately	1.108 (0.891, 1.379)	0.357	1.393 (1.028, 1.889)	0.033
More exposed	1	1	1	1
**Child’s sex**	**		***	
Male	1	1	1	1
Female	1.179 (1.004, 1.383)	0.044	1.482 (1.22, 1.798)	0.000
**Child's health**	**		***	
Sick	1	1	1	1
Not ill	1.179 (1.006, 1.416)	0.042	1.801 (1.452, 2.234)	0.000
**Desirability of the child**	ns		ns	
Desired	0.934 (0.79, 1.104)	0.422	0.994 (0.808, 1.223)	0.957
Not desired	1	1	1	1

Explanatory note: *** = Significant at the 1% level; **= Significant at the 5% level; *= Significant at the 10% level; ns = Not significant

### Region of residence

Compared to children in the North, children in the East and West regions were 0.54 (95% CI 0.31, 0.97 and 0.65 (95% CI 0.47, 0.89) times less likely to experienced cessation of EBF before six months, respectively. In contrast, children in Abidjan were about two times more likely to be deprived of exclusive breastfeeding than children in the North. The difference in risk of discontinuing EBF was not significant between children in the North and those in the Central and Southern regions without Abidjan. Thus, exclusive breastfeeding is practiced over a relatively long period of time in the East and West regions than in the Center, North and South regions (with Abidjan).

In models M1 to M14, region of residence remained significant, whereas it was not significant in the crude effects model. The coefficients associated with its modalities changed with the introduction of variables such as religion, household standard of living, cohabitation of spouses, level of education, age at delivery, and degree of exposure to medical discourse on breastfeeding and the media. Therefore, region of residence primarily affects the duration of exclusive breastfeeding indirectly through the other variables. In other words, some of the differences in risk of EBF discontinuation associated with the mother’s region of residence are explained by differences in standard of living, education, and exposure to medical discourse on breastfeeding. In addition, the city of Abidjan became significant with the inclusion of standard of living in the M5 model and decreased from 5 to 1% from the M11 model onwards with the introduction of mothers’ media exposure, then remained stable until the final model. The influence of the city of Abidjan is mediated by household standard of living and women’s media exposure.

### Health status of the child

The risk of being deprived of exclusive breastfeeding among children who were not sick in the two weeks prior to the survey was greater than among children who had been sick (adjusted OR 1.8; 95% CI 1.452, 2.234). Among children who showed signs of cough, diarrhea, or fever, the duration of EBF tended to be longer than if they were healthy. In both the crude and final models, the health status of the child had a significant influence on the duration of the exclusive breastfeeding of children before their 6th month.

### Household standard of living

The duration of exclusive breastfeeding is significantly influenced by the household’s level of economic well-being. According to the Table 4, children from households classified in the very low and low standard of living quintile more likely (adjusted OR 1.59; 95% CI 0.95, 2.65 and adjusted OR 2.15; 95% CI 1.33, 3.5, respectively) to be deprived of EBF than children from households classified in the very high standard of living quintile. Children from households classified in the middle and high standard of living quintile are 1.85 (95% CI 1.19, 2.87) and 2.53 times (95% CI 1.69, 3.8) more likely to be deprived of exclusive breastfeeding before six months, respectively.

While the interaction between EBF and this variable was insignificant in the crude model, its significance appeared in the model (M5). Thus, its influence on exclusive breastfeeding cessation is indirect and reinforced by other variables. Moreover, the effect of the “Very weak” modality fades with the introduction of the “Exposure to medical discourse” variables. This could be because mothers in households with very low economic power do not have access to health services to the same degree as mothers living in households with very high economic power. This could result in some inequality in access to breastfeeding information.

### Sex of the child

The sex of the child significantly influences the duration of the exclusive breastfeeding. Indeed, the risk of being deprived of EBF before six months is higher for girls (adjusted OR 1.48; 95% CI 1.22, 1.80) than for boys. Thus, girls are less likely to be exclusively breastfed than boys.

### Exposure to medical speech

Children of mothers with high exposure to medical discourse were less likely (adjusted OR 0.74; 95% CI 0.60, 0.91) to be denied exclusive breastfeeding before six months than those whose mothers had low exposure. Otherwise, children of mothers who had at least four antenatal cares and were attended by skilled health personnel at delivery were more likely to receive EBF than other children. The adjusted OR associated with unexposed (adjusted OR 1.36; 95% CI 0.87, 2.13) and highly exposed (adjusted OR 0.74; 95% CI 0.59, 0.91) mothers show that increasing the degree of exposure to medical speech increases the odds of exclusive breastfeeding.

### Mother’s level of education

The mother’s education level is relevant to explain the duration of exclusive breastfeeding. Children of uneducated mothers and those of mothers with primary education are about 0.73 (95% CI 0.52, 1.02) and 0.64 (95% CI 0.45, 0.91) times less likely to be deprived of exclusive breastfeeding, respectively. Women with no education and those with primary education practice exclusive breastfeeding longer than other women.

In models M7 to M12, the mother’s education level had no influence on the dependent variable. The effect of this variable on the continuation or cessation of exclusive breastfeeding became significant with the introduction of the child’s health status in model M13. The influence of this variable is therefore boosted by the child’s health status.

## Discussion

The present study analyzed explanatory factors of exclusive breastfeeding among children aged 0 to 5 months, based on the 2016 MICS-5 database from Cote d’Ivoire.

Our results showed that duration of EBF varies significantly associated with region of residence. Influence of this variable is indirect and is mediated by several factors that affect behavior of breastfeeding women. In the literature, the “region of residence” variable is increasingly being abandoned in favor of variables such as “rural” and “urban”, district or neighborhood, which represent smaller living areas and could therefore have an immediate impact on behavior. However, our results are in agreement with those of the Nigerian studies [15] and Bangladesh [16] which showed significant disparities in the practice of exclusive breastfeeding between regions.

We found that in children with signs of coughing, diarrhea or fever, breastfeeding tends to be prolonged. This association may reflect a reverse causality that was observed by Marquis et al. [17]. Their research found that poor child growth and health led to increased breastfeeding.

This result is contrary to that of Vilain [18], which showed that the probability of continuing breastfeeding beyond 10 weeks was lower in sick children. Nevertheless, this result could be explained by the fact that mothers are more attentive to certain recommendations that encourage them to continue exclusive breastfeeding despite the disease, while also providing oral rehydration solutions or local preparations if necessary [8].

In contrast with our results, studies have shown a link between the decline of exclusive breastfeeding and improvements in household living standards [19, 20]. However, our results are consistent with a Nigerian study that found that women from poorer households were more likely to interrupt exclusive breastfeeding than their counterparts from wealthier households [15, 21]. This could be explained by the fact that women from wealthy backgrounds usually turn to qualified health professionals or have easier access to quality information advocating EBF through media. For working-class women, they turn first to their close relatives (mother, mother-in-law, etc.) or to their neighbors. However, specialist interlocutors are more favorable and unanimous in their support of exclusive breastfeeding than reference persons (parents and neighbors), who rely on their more or less successful experience [22].

In contrast to other studies in Kenya [23], in Nigeria [15] and in Vietnam [19] that found that male newborns were introduced to complementary foods early compared to girls, our study shows that male infants are more likely to be exclusively breastfed than girls. This finding is consistent with others studies in Egypt and in Timor-Leste [24, 25]. Our result could be explained by a culturally related male gender preference for breastfeeding, feeding, and health care. However, the gender difference in breastfeeding practices warrants further investigation to understand the sociocultural factors that leading to this difference.

antenatal cares play an important role in mothers’ decision-making about EBFs because pregnant women receive counseling from healthcare staff there [26]. Previous studies have shown that the rate and duration of breastfeeding was better in women who performed four or more antenatal cares [19, 24, 27–29]. Indeed, the assistance of health professionals during pregnancy would encourage changes in attitudes and practices towards exclusive breastfeeding from birth [30].

In this study, women with primary education practiced exclusive breastfeeding longer than women with secondary education or higher. This result is consistent with those of Asfaw et al. in Debre Berhan District, Ethiopia [31], of Asare [32] and Hossain et al. [16] in Bangladesh. This could be because educated mothers have better employment opportunities than their uneducated counterparts. Therefore, they find it difficult to maintain the practice of EBF for up to six months due to lack of time and other factors related to the job they are doing [16, 31]. However, for some authors, mother’s education level would have a positive influence on the timely initiation of exclusive breastfeeding, its duration and the avoidance of prelacteal feeding [33, 34]. Also, some relatively recent studies [28–30, 35] are favorable to the idea that educated mothers are more likely to have better access to and use of information than mothers with low levels of education.

In this study, variables such as residence, religion, maternal age, spousal cohabitation, parity, and child desirability had no statistically significant relationship with the practice of exclusive breastfeeding. Compared with other studies, rural [31], have more than two children [28] and be over 25 years old [31, 36] were positive factors in the practice of exclusive breastfeeding.

### Limitations of the study

The main limitations of our study are that we used secondary data and information on breastfeeding was collected based on the 24-hour recall, as this approach does not capture the age at which the child stopped being exclusively breastfed. The absence of this variable forced us to consider only the survey date as the end date of observation. As a result, we could not adequately capture all cases of right truncation in the estimation of actuarial survival curves (at the bivariate descriptive analysis level). In addition, the variables relating to the spouse or partner, the socialization environment, the influence of contradictory information on breastfeeding, the choice of the mode of feeding of the child during pregnancy, the early initiation of breastfeeding and the occupation of the mother are not considered in the present study because of certain technical difficulties for some of them and unavailability for others.

## Conclusions

The prevalence of exclusive breastfeeding in Cote d’Ivoire was one of the lowest in West Africa in 2021, with large regional disparities and a strong influence of socioeconomic and cultural conditions. To improve the level of exclusive breastfeeding practice, the government and its partners should facilitate access to prenatal health services and deliveries in health centers for all social strata of women under the assistance of qualified persons. They should also build the capacity of health professionals to counsel and assist mothers in adhering to the practice of exclusive breastfeeding and then to support them in continuing the practice until the recommended time, regardless of the health status and sex of the child.

## Data Availability

The datasets used and/or analyzed during the current study are available on the MEASURE DHS / ICF International website: [18]
